# DNA Methylation Analysis of Ribosomal DNA in Adults With Down Syndrome

**DOI:** 10.3389/fgene.2022.792165

**Published:** 2022-04-27

**Authors:** Francesco Ravaioli, Michele Zampieri, Luca Morandi, Chiara Pirazzini, Camilla Pellegrini, Sara De Fanti, Noémie Gensous, Gian Luca Pirazzoli, Luisa Sambati, Alessandro Ghezzo, Fabio Ciccarone, Anna Reale, Daniela Monti, Stefano Salvioli, Paola Caiafa, Miriam Capri, Alexander Bürkle, Maria Moreno-Villanueva, Paolo Garagnani, Claudio Franceschi, Maria Giulia Bacalini

**Affiliations:** ^1^ Department of Experimental, Diagnostic and Specialty Medicine (DIMES), University of Bologna, Bologna, Italy; ^2^ Department of Experimental Medicine, Sapienza University of Rome, Rome, Italy; ^3^ Functional and Molecular Neuroimaging Unit, IRCCS Istituto Delle Scienze Neurologiche di Bologna, Bologna, Italy; ^4^ Department of Biomedical and Neuromotor Sciences, University of Bologna, Bologna, Italy; ^5^ IRCCS Istituto Delle Scienze Neurologiche di Bologna, Bologna, Italy; ^6^ Department of Biological, Geological and Environmental Sciences, University of Bologna, Bologna, Italy; ^7^ Interdepartmental Centre Alma Mater Research Institute on Global Challenges and Climate Change, University of Bologna, Bologna, Italy; ^8^ Department of Internal Medicine and Clinical Immunology, CHU Bordeaux (Groupe Hospitalier Saint-André), Bordeaux, France; ^9^ UMR/CNRS 5164, ImmunoConcEpT, CNRS, University of Bordeaux, Bordeaux, France; ^10^ Medical Department, Maggiore Hospital, Bologna, Italy; ^11^ IRCCS Istituto Delle Scienze Neurologiche di Bologna, U.O.C. Clinica Neurologica Rete Neurologica Metropolitana (NEUROMET), Bologna, Italy; ^12^ DIMES, School of Medicine, University of Bologna, Bologna, Italy; ^13^ IRCCS San Raffaele Roma, Department of Human Sciences and Promotion of the Quality of Life, San Raffaele Roma Open University, Rome, Italy; ^14^ Department of Experimental and Clinical Biomedical Sciences “Mario Serio”, University of Florence, Florence, Italy; ^15^ Department of Cellular Biotechnologies and Haematology, Sapienza University of Rome, Rome, Italy; ^16^ Molecular Toxicology Group, Department of Biology, University of Konstanz, Konstanz, Germany; ^17^ Applied Biomedical Research Center (CRBA), S. Orsola-Malpighi Polyclinic, Bologna, Italy; ^18^ CNR Institute of Molecular Genetics “Luigi Luca Cavalli-Sforza”—Unit of Bologna, Bologna, Italy; ^19^ Department of Laboratory Medicine, Clinical Chemistry, Karolinska Institutet, Karolinska University Hospital, Huddinge, Sweden; ^20^ Laboratory of Systems Medicine of Healthy Aging, Department of Applied Mathematics, Lobachevsky University, Nizhny Novgorod, Russia

**Keywords:** Down syndrome, ribosomal genes, rDNA, aging, DNA methylation

## Abstract

Control of ribosome biogenesis is a critical aspect of the regulation of cell metabolism. As ribosomal genes (rDNA) are organized in repeated clusters on chromosomes 13, 14, 15, 21, and 22, trisomy of chromosome 21 confers an excess of rDNA copies to persons with Down syndrome (DS). Previous studies showed an alteration of ribosome biogenesis in children with DS, but the epigenetic regulation of rDNA genes has not been investigated in adults with DS so far. In this study, we used a targeted deep-sequencing approach to measure DNA methylation (DNAm) of rDNA units in whole blood from 69 adults with DS and 95 euploid controls. We further evaluated the expression of the precursor of ribosomal RNAs (RNA45S) in peripheral blood mononuclear cells (PBMCs) from the same subjects. We found that the rDNA promoter tends to be hypermethylated in DS concerning the control group. The analysis of epihaplotypes (the combination of methylated and unmethylated CpG sites along the same DNA molecule) showed a significantly lower intra-individual diversity in the DS group, which at the same time was characterized by a higher interindividual variability. Finally, we showed that RNA45S expression is lower in adults with DS. Collectively, our results suggest a rearrangement of the epigenetic profile of rDNA in DS, possibly to compensate for the extranumerary rDNA copies. Future studies should assess whether the regulation of ribosome biogenesis can contribute to the pathogenesis of DS and explain the clinical heterogeneity characteristic of the syndrome.

## Introduction

Down syndrome (DS), the most frequent chromosomal disorder in live births, is caused by the complete or partial trisomy of chromosome 21 (HSA21). DS is a multisystemic condition, whose phenotypic traits include characteristic craniofacial features, neurological complications and cognitive impairment, heart, developmental defects, and immune system abnormalities (Bull 2020). Persons with DS undergo an atypical aging (Zigman 2013) and show clinical and molecular features characteristic of older adults (Carfi et al., 2014; Horvath et al., 2015; Conte et al., 2019; Franceschi et al., 2019; Gensous et al., 2020a); thus, this condition has been considered among progeroid syndromes (Martin and Oshima, 2000). The molecular pathogenesis of these phenotypes, which vary greatly in presentation and severity, is complex and only partially understood. Omic analyses have highlighted profound alterations at the epigenetic (Bacalini et al., 2014a; Yu et al., 2020; Muskens et al., 2021), transcriptomic (Costa et al., 2011; Antonaros et al., 2021), proteomic (Liu et al., 2017; Sullivan et al., 2017) and glycomic (Borelli et al., 2015) level. Possibly, the syndrome results from the concomitant action of two mechanisms: a dosage effect of genes located on HSA21 and a nonspecific global alteration of cellular homeostasis due to the extra copy of HSA21 (Antonarakis et al., 2020).

**FIGURE 1:**
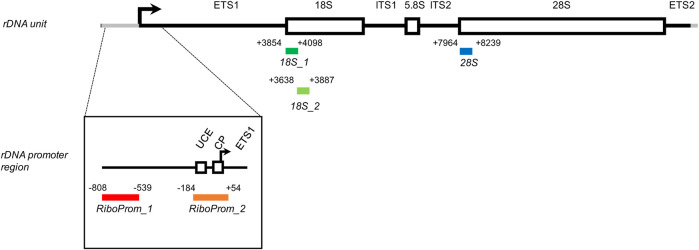
Structure of the rDNA unit and localization of the target regions assessed in this study**.** Each amplicon included in the targeted-bisulfite sequencing assay is encoded by its coordinates concerning the rDNA unit (hg38, chr21:8292347–8222335). RiboProm_1 (red, from -808 to -539) is located on a distal promoter of the rDNA unit. RiboProm_2 (orange, from -184 to +54) is located on the proximal promoter of the rDNA unit and encompasses the upstream control element (UCE), the core promoter (CP), and the transcription starting site (indicated by the black arrow). 18S1 (dark green, from +3854 to +4098) and 18S2 (light green, from + 3638 to + 3887) are located onto the 5′ end of the 18S gene. 28S (dark blue, from + 7964 to 8239) is located on the 5′ end of the 28S gene. ETS: external transcribed spacer; ITS: internal transcribed spacer.

Among the genes located on HSA21, there are those encoding ribosomal RNAs (rRNAs) which are organized in arrayed clusters of tandem repeats that are part of the nucleolar organizer regions (NORs) (Raška et al., 2004). Each repeated unit encodes for a 45S pre-ribosomal RNA (RNA45S) that serves as the precursor for 18, 5.8, and 28S rRNAs (Figure 1). A variable number of 30–40 rDNA repeats is located on the short arm of the five acrocentric chromosomes (HSA13, HSA14, HSA15, and HSA22 in addition to HSA21), for a total of about 400 rDNA copies in diploid cells. rDNAs are critical housekeeping genes (Kobayashi 2011) as their transcription by RNA polymerase I consumes the majority of cellular energy and is a limiting step for ribosome biogenesis. The expression of rDNA loci is tightly regulated during development and in response to nutrient availability, growth factors, and other intra and extracellular stimuli (James and Zomerdijk, 2004; Schlesinger et al., 2009; Vizoso-Vázquez et al., 2018). In mammals, rDNA copies classify into three distinct activity states: silent, inactive, and active (Kresoja-Rakic and Santoro 2019). DNA methylation (DNAm) of the promoter occurs in silent rDNAs, which have constitutive heterochromatic features. On the contrary, both inactive and active rDNAs are not methylated at the promoter. Inactive units are nucleosome-packed at the coding region and are not transcribed, while active units are loosely packed and actively transcribed, expressing the RNA45S precursor. As a result of this complex regulation, in somatic cells, only about 50% of the available rDNA units are actually transcribed (Schlesinger et al., 2009).

The rDNA cluster of HSA21 is located on the short arm of the chromosome, and it is therefore not included in the DS critical region. However, most cases of DS present with a complete trisomy of HSA21 and *de facto* have 30–40 additional copies of the rDNA unit. Given the central role of ribosome biogenesis regulation in cellular functions and homeostasis, it can be suggested that this extra rDNA content can have a role in DS pathogenesis (Demirtas 2009). Only few studies investigated this aspect so far, mainly measuring ribosome biogenesis by the AgNOR procedure, which consists in the silver staining of a set of acidic and argyrophilic non-histone proteins that are associated with actively transcribed NOR (Imamoglu et al., 2005; Imamoglu et al., 2006; Yilmaz and Demirtas 2008; Imamoglu et al., 2016; Lyapunova et al., 2017; Penzo et al., 2019). To the best of our knowledge, no study has focused on the factors that can regulate transcription of the rDNA locus, such as DNAm.

Despite the importance of DNAm in the regulation of rDNA transcription, there is a paucity of studies that evaluated this epigenetic modification. In humans, each rDNA unit harbors more than 1,500 CpGs, but these sites are excluded from microarrays and are usually filtered out in bioinformatic analyses of whole-genome bisulfite sequencing data. Targeted approaches have been used to study rDNA methylation. Most of these studies focused on cancer (Shao et al., 2021), while others evaluated rDNA methylation changes in aging, age-related neurodegenerative diseases (e.g., Alzheimer’s disease) (Faria et al., 2020), and progeroid conditions (Machwe et al., 2000).

In the present study, we described rDNA methylation profiles measured in whole blood from adults with DS and age, sex-matched, and euploid controls using a targeted deep-sequencing approach. We further evaluated the expression of the RNA45S precursor in peripheral blood mononuclear cells (PBMCs) from the same subjects.

## Methods

### Samples

The samples analyzed in this study were collected from Italian persons with DS and euploid subjects, recruited in the framework of an Italian project on intellectual disability supported by the CARISBO Foundation and of the MARK-AGE project, funded by the European Union’s Seventh Framework Program. Details on both studies have been previously described (Ghezzo et al., 2014; Baur et al., 2015; Bürkle et al., 2015; Capri et al., 2015). The studies were approved by the local Ethical Committee (S. Orsola Hospital, University of Bologna; ethical clearance documents #126/2007/U/Tess, #75/2008/U/Tess and following amendments). Written informed consent to participate in the study was obtained from adult persons with DS and healthy subjects and from parents or authorized tutors for those under age. Written informed consent was also obtained for adult DS persons from parents or relatives. In both CARISBO and MARK-AGE projects, whole blood from 69 persons with DS and 95 euploid subjects was collected in EDTA tubes. In addition, in the framework of the MARK-AGE project, PBMCs were also isolated from 49 persons with DS and 33 controls as previously described (Moreno-Villanueva et al., 2015). For 42 persons with DS and 29 euploid controls, both whole blood and PBMC samples were available. Karyotype information was available for 41 persons with DS; of these, 35 were HSA21 trisomy, eight were mosaics, and one was a translocation. As previously described (Ghezzo et al., 2014), persons with DS underwent clinical and neuropsychological function evaluation using the following tests: WISC-III, WAIS-R, Spatial Span, Categorical fluency, Tower of London, Token test, Frontal Assessment Battery (FAB), the Visual Object and Space Perception Battery (VOSP), Vineland Adaptive Behavior Scales (VABS), and DSQIID Questionnaire (Dementia Screening Questionnaire for Individuals with Intellectual Disabilities).

### Sample Extraction and Processing

DNA was extracted from whole blood samples using the Qiamp DNA Mini Kit (Qiagen, Hilden, Germany) following the manufacturer’s protocol. Then, 500 ng of DNA was bisulfite-converted using the EZ-96 DNA Methylation Kit as indicated by the manufacturer. Extraction of RNA from PBMCs and retrotranscription were previously described (Ciccarone et al., 2018). Briefly, total RNA was isolated from PBMCs using the RNeasy Mini Kit (Qiagen, Hilden, Germany) and converted to cDNA using the SuperScript VILO cDNA Synthesis Kit (Invitrogen, Waltham, MA, United States).

Standard curves were prepared using universal methylated and universal unmethylated DNA (Millipore, Burlington, MA, United States) that were combined in order to generate standards at 0, 25, 50, 75, and 100% DNAm levels. Each point of the curve was sequenced in triplicate.

### Construction of Libraries for Target Sequencing

To analyze the DNAm of rDNA genes, a targeted-bisulfite sequencing approach was adopted (Figure 1). Bisulfite-specific primers for the rDNA promoter (RiboProm_1 and RiboProm_2) were previously published (Flunkert et al., 2018). Primers mapping at the 5′ of 18S and 28S target regions were designed using MethPrimer 2.0 (Supplementary Table S1). Forward and reverse primers were added at each 5′ end with Nextera™ adapter sequences TCG​TCG​GCA​GCG​TCA​GAT​GTG​TAT​AAG​AGA​CAG and GTC​TCG​TGG​GCT​CGG​AGA​TGT​GTA​TAA​GAG​ACA​G, respectively. In addition, a random nucleotide spacer (N) was included between Illumina adapters and primers in order to increase sequence variability. The sequences of the 5*’* end primer pairs used are reported in Supplementary Table S1. Sequencing libraries were generated through a two-step PCR approach. Briefly, in the first step of PCR, 5 ng of bisulfite-converted DNA was amplified using Phusion U (ThermoFisher, Waltham, MA, United States) added with 1M Betaine (Merk, Darmstadt, Germany), 150 nM forward and reverse primers, 1.75 mM MgCl2 (Agena Bioscience, San Diego, CA, United States), and 200 μM dNTP (ThermoFisher, Waltham, MA, United States). Thermal cycler conditions were set as follows: 1x cycle at 95°C for 1′ 40’’; 1x cycle at 98°C for 1’; 1x cycle at 58°C for 2’; 1x cycle at 72°C for 1’; 36 cycles at 98°C for 10″, 58°C for 40″, 72°C for 20’’; 1x cycle at 72°C for 5’; and hold at 4°C. Amplicons were pooled sample-wise and purified using MagSi-NGS plus beads (MagTivio BV, Nuth, The Netherlands) as indicated by the manufacturer’s protocols. In the second step of PCR, 10 μL of pooled samples was indexed using Illumina Nextera XT Index Set A as indicated in the Nextera Library Prep Guide. The indexed libraries were then purified and normalized before sequencing as indicated in the Nextera Library Prep Guide. Sequencing was performed with a Micro V2 300 PE reagent kit on an Illumina MiSeq System.

### EpiTYPER Assay

The EpiTYPER assay (Agena, San Diego, CA, United States) was used as an alternative technique to analyze DNAm of the rDNA locus, as previously described (Bacalini et al., 2014b; Gensous et al., 2020b). The same target regions evaluated by targeted-bisulfite sequencing were PCR-amplified using the following primers: Ribo forward: AGG​AAG​AGA​GGT​GTG​TTT​TGG​GGT​TGA​TTA​GAG; Ribo reverse: CAG​TAA​TAC​GAC​TCA​CTA​TAG​GGA​GAA​GGC​TAA​AAC​CCA​ACC​TCT​CCA​AC; 18S forward: AGG​AAG​AGA​GGT​TTG​TTG​TTT​TTT​TTG​GAT​GTG​G; 18S reverse: CAG​TAA​TAC​GAC​TCA​CTA​TAG​GGA​GAA​GGC​TCC​TTA​CCT​ACC​TAA​TTA​ATC​CTA​CCA​A; 28S forward: AGG​AAG​AGA​GGG​TAT​TTA​GTT​TTA​GAT​GGA​GTT​TAT​TAT​T; 28S reverse: CAG​TAA​TAC​GAC​TCA​CTA​TAG​GGA​GAA​GGC​TAA​AAA​AAA​CTA​ACC​AAA​ATT​CCC. The EpiTYPER assay returns the methylation of single CpGs or of small groups of adjacent CpGs (CpG units) depending on the target sequence. The EpiTYPER assay was applied to 47 persons with DS and 33 euploid controls from the above-described cohort.

### Data Handling

Paired-end reads obtained from Illumina MiSeq were quality-checked using *FastQC* (https://www.bioinformatics.babraham.ac.uk/projects/fastqc/). Adapter sequences were trimmed using *cutadapt* (M. Martin 2011) and finally, paired-end reads were merged using the *PEAR* tool (Zhang et al., 2014), with a minimum of 20 overlapping residues and a maximum read length of 450. FASTQ assembled reads were converted to FASTA using the *seqtk* tool (https://github.com/lh3/seqtk). All read handling and processing tools were compiled in Anaconda2-based environments.

To analyze the DNAm status for each target sequence, the *AmpliMethProfiler* Analysis Pipeline was followed (Scala et al., 2016). Briefly, this pipeline was designed to analyze deep bisulfite sequencing data for the given genomic regions. For each sample, AmpliMethProfiler filters target-specific reads with a mean quality score (Phred) > 33 and performs target-specific, template alignment filtering reads with length >80% of the reference sequence. Finally, it calculates the DNAm status at each cytosine within the CpG dinucleotide framework. For each sample, the *AmpliMethProfiler* pipeline generates several outputs including a file containing the DNAm value of each CpG site calculated as a percentage of all the reads for the considered target region and a file containing the DNAm profile for each read mapping to the target region. Sequencing coverage was calculated for each target region, and samples with coverage <100 were excluded from further analyses. After filtering, mean coverage was 1,583 (228–3884) for RiboProm_1; 1,416 (227–3122) for RiboProm_2; 2252 (596–5214) for 18S1; 2732 (297–6212) for 18S2, and 2527 (791–5404) for 28S2.

### Gene Expression Analysis

Expression of the *RNA45S* precursor and of the reference gene (β-glucuronidase, *GUSB*) was measured by real-time PCR using Taqman Gene Expression Assays (Applied Biosystems, Monza, Italy) on the iCycler IQ detection system (Bio-Rad, Hercules CA). An internal control (cDNA prepared from MCF7 cells) was used as a calibrator among the different runs. Expression values were calculated using the ΔΔCt method.

### Data Analysis

DNAm was compared between the groups under investigation by the ANOVA test including age, sex, and batch (Model 1) or age, sex, batch, and coverage (Model 2) as covariates. The association between DNAm and age in DS and control groups was calculated by the linear model using sex and batch as covariates. The differences in DNAm variability between DS and CTRL groups were calculated using the *varFit* function compiled in the *missMethyl* R package (Phipson et al., 2016) using age, sex, and batch as covariates. Nominal *p*-values were corrected for multiple testing using the Benjamini–Hochberg (BH) method.

An AmpliMethProfiler analysis pipeline was designed to determine the DNAm status of each CpG site at a single-DNA molecule level. This pipeline allows to analyze how methylated and unmethylated CpGs organize along the target regions. Each possible combination is defined as an epihaplotype. However, as the number of possible epihaplotypes depends on the number of CpG dinucleotides included in each target sequence, we observed that the number of expected epihaplotypes largely surpasses our sequencing depth. Therefore, we filtered out epihaplotypes occurring only one time in each sample. In addition, the samples with a total coverage of less than 1,000 reads were removed to facilitate the calculation of alpha-diversity index rarefaction curves. Filtered epihaplotype frequency tables were inputted for the analysis of alpha-diversity *via* a *qiime*-based pipeline included in *AmpliMethProfiler*. Briefly, for alpha diversity, *AmpliMethProfiler* calculates the Shannon diversity index. The differences between alpha-diversity indexes in DS and control groups were measured onto the rightmost shared point of the rarefaction curves using the ANOVA test, including sex, age, and batch as covariates. Finally, *p*-values were FDR-corrected using the BH approach.

The differences in *RNA45S* gene expression between DS and control groups were determined by the ANOVA test correcting for age and sex. Association with age was calculated by the linear model using sex as a covariate. Finally, DNA methylation of each CpG site assessed by the assay was correlated with the expression of the RNA45S precursor using Pearson’s correlation, and *p*-values were corrected for multiple tests by the BH approach.

## Results

### Analysis of rDNA Methylation by Targeted-Bisulfite Sequencing

To evaluate rDNA methylation, we performed bisulfite sequencing of five target regions across the rDNA unit (Figure 1).

*RiboProm_1* and *RiboProm_2* targets were designed as indicated in previous studies (Flunkert et al., 2018). *RiboProm_1* is located at a distal rDNA promoter, whereas *RiboProm_2* encompasses the rDNA upstream control element (UCE), the core promoter (CP) and the transcription starting site. *18S_1*, *18S_2*, and *28S* targets were designed to cover the 5’ end of their relevant rRNA sequences as described in previous studies (Bacalini et al., 2014a; Gensous et al., 2020a). *RiboProm_1*, *RiboProm_2*, *18S_1*, *18S_2*, and *28S*, respectively, include 37, 26, 27, 13, and 30 CpG sites, whose DNAm is measured at single-base resolution. Preferential amplification of bisulfite-converted DNA depending on its original DNA methylation status (a phenomenon called PCR-bias) has been reported for some genomic regions (Warnecke et al., 1997). To check for PCR bias in our assays, we processed samples at known DNAm percentages (0, 25, 50, 75, and 100%) and analyzed the correlation between observed and expected DNAm values. For the large part of CpGs assayed, we observed a strikingly significant correlation (r Pearson >0.96; *p*-value<0.01) between the observed and expected values, confirming that our rDNA methylation assay is quantitative (Supplementary Figure S1). The assay was applied to DNA extracted from the whole blood of 69 persons with DS and 95 euploid, age, and sex-matched control subjects (CTRL) (Table 1).

**TABLE 1:** Characteristics of the cohort.

DNAm				RNA45S expression			
n°		CTRL	DS	n° (overlapping with the DNAm cohort)		CTRL	DS
		95	69			33 (29)	49 (42)
Age				Age			
	*Min*	12.5	10.6		*Min*	35.1	19
	*Max*	82.8	70.1		*Max*	66	68
	*Mean (SD)*	43.9 (15.3)	34.3 (14.4)		*Mean (SD)*	46.3 (9.3)	40.6 (12.1)
Sex				Sex			
	*Male (%)*	*36 (37.9%)*	36 (52.1)		*Male (%)*	*17 (51.5%)*	27 (55.1)
	*Female (%)*	*59 (62.1%)*	33 (47.8)		*Female (%)*	*16 (48.5%)*	22 (44.9)

Min, minimum value; Max, maximum value; Mean, mean value; SD, standard deviation.

Surprisingly, during the quality assessment of the experiment, we found a positive correlation between DNAm and sequencing coverage for all the target regions (Supplementary Figure S2). Based on the results of the standard curves, it is unlikely that this correlation is due to a PCR-amplification bias depending on the original DNA methylation status (see *Discussion*). In addition, we did not observe significant differences in coverage between DS and CTRL, with the exception of 18S_2 amplicon (p-value=0.03; Supplementary Figure S3). Notwithstanding, coverage was evaluated as a potential confounding effect in statistical analyses, as described below.

### Trend Toward rDNA Hypermethylation in Persons With DS

**FIGURE 2:**
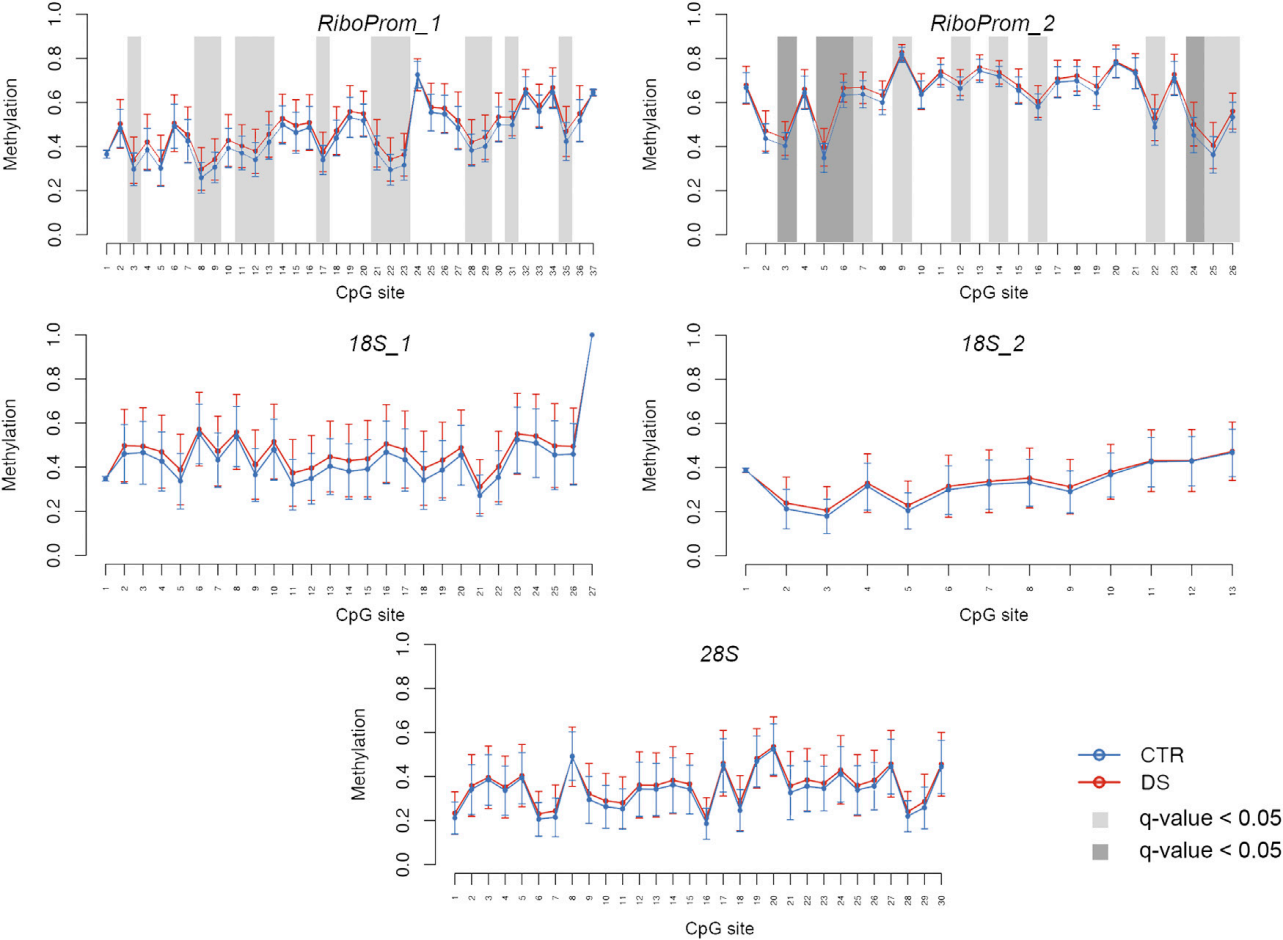
DNA methylation profiles of rDNA target regions in persons with DS. For RiboProm_1, RiboProm_2, 18S_1, 18S_2, and 28S target regions, the plots show mean DNA methylation and standard deviation in persons with DS and controls. Differential methylation was calculated using ANOVA and correcting for age, sex, and batch. CpGs with q.value < 0.01 are highlighted with dark gray boxes; CpGs with q-value < 0.05 are highlighted with light gray boxes.

For each target region, we compared DNAm levels between DS and CTRL, correcting for potential confounding factors (Model 1: age, sex, and experimental batch; Model 2: age, sex, experimental batch, and coverage) (Figure 2; Supplementary Table S2).

In *RiboProm_1* and *RiboProm_2*, we found multiple CpGs significantly hypermethylated in DS, both at the nominal level (*p*-value<0.05) and after FDR correction (q-value<0.05), according to Model 1. Statistical significance was confirmed also when coverage was included as a covariate (Model 2, Supplementary Table S2). We found the highest significance in a region in *RiboProm_2* amplicon encompassing CpG5–CpG9 (q-values ranging from 0.002 to 0.01, Supplementary Table S2), which showed an average hypermethylation of 3% in DS.

A trend toward hypermethylation in DS was also evident in *18S_1*, *18S_2*, and *28S*, although a smaller fraction of CpGs reached statistical significance (*p*-value<0.05) in these regions (Supplementary File S2).

Within the group of DS, the assessed target regions did not show DNAm differences according to karyotype (complete trisomy, mosaicism, or translocation; data not shown).

An alternative approach to measure DNAm, the EpiTYPER assay was used to validate the observed differences between DS and CTRL in a subset of samples. The EpiTYPER analysis showed a trend toward rDNA hypermethylation in DS compared to CTRL that reached statistical significance (q-value <0.05) for some CpG units, thus confirming the results generated by targeted-bisulfite sequencing (Supplementary Figure S4).

**FIGURE 3:**
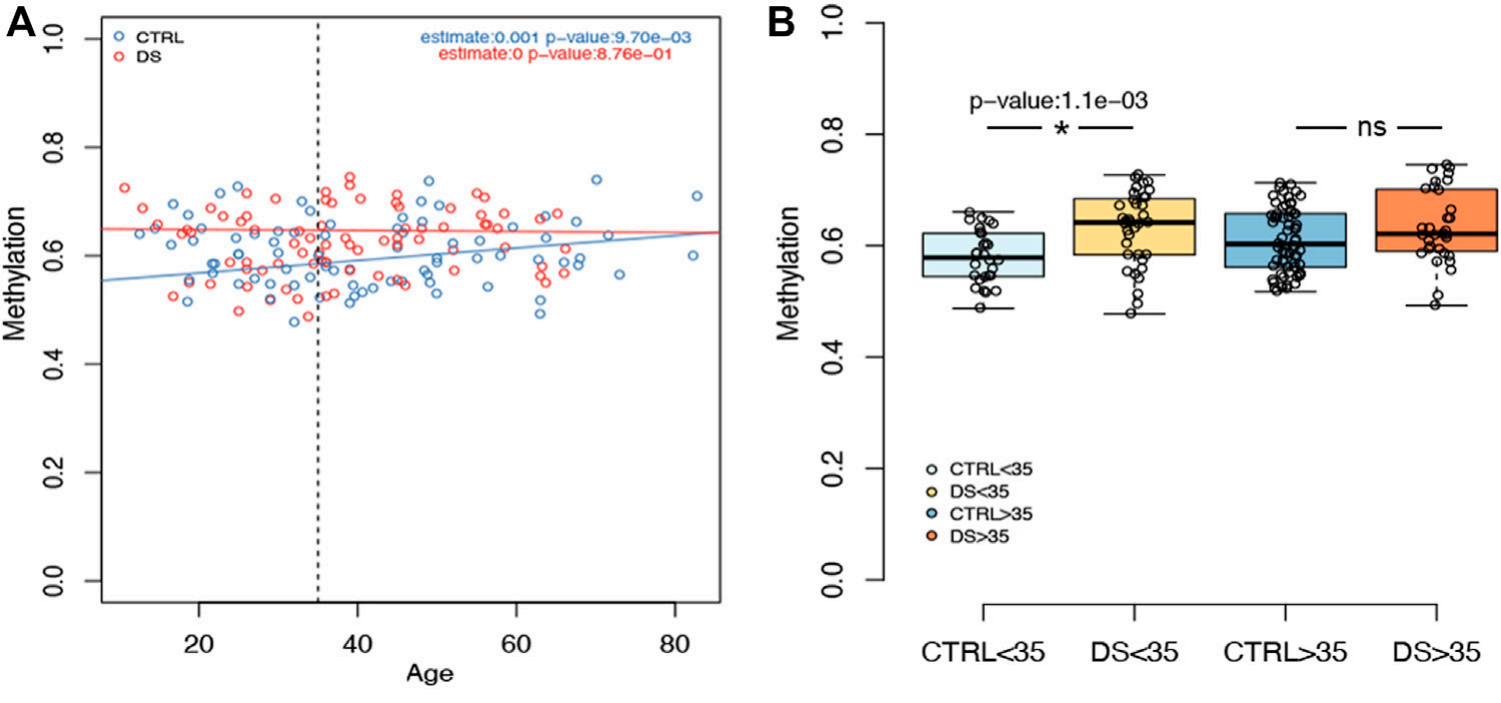
Examples of rDNA methylation values according to age. **(A)** For CpG 8 within RiboProm_2 amplicon, the boxplot reports the association between DNAm and age in persons with DS and controls. Estimate and *p*-values were calculated, respectively, by a linear model and by ANOVA correcting for age, sex, and batch. **(B)** For the same CpG site, the boxplots highlight DNAm differences between DS and control groups stratified by age (threshold = 35 y.o). *P*-values were calculated using an ANOVA test after correcting for age, batch, and sex.

We then analyzed the association between rDNA methylation and age in DS and control groups separately, correcting for sex and experimental batch (Supplementary Table S2). In the control group, we observed significant hypermethylation with age in a subset of CpG sites included in *RiboProm_1* and *RiboProm_2* amplicons (*p*-value < 0.05, Supplementary Table S2). The strongest associations were found for the group of CpG sites encompassing CpG6–CpG9 within *RiboProm_2* (*p*-values ranging from 0.002 to 0.041, Supplementary Table S2), which also showed the most significant differences between persons with DS and controls, as discussed above. On the contrary, no significant association with age was found in the DS group. In Figure 3A, the association between *RiboProm_2* CpG8 methylation and age (estimate = 0.001, *p*-value = 0.01 in CTRL; not significant in DS) is reported as a representative example.

Consistently, when we divided the cohort according to age, we observed that DNAm differences in *RiboProm_1* and *RiboProm_2* were more pronounced between young (≤35 y.o.) persons with DS and controls than between older individuals (>35 y. o.) (Supplementary Table S2). Figure 3B reports DNAm values for *RiboProm_2* CpG8 dividing the groups according to age, highlighting a significant difference in younger DS *vs* CTRL subjects (*p*-value = 0.001) but not in the older ones (Supplementary Table S2).

For *18S_1*, *18S_2*, and *28S* targets, we found few CpG sites showing nominally significant association (*p*-value<0.05) of DNAm with age. Similarly, these targets showed limited DNAm difference between DS and control groups when subjects were stratified by age.

### Decreased DNAm Epihaplotype Diversity Within DS

**FIGURE 4:**
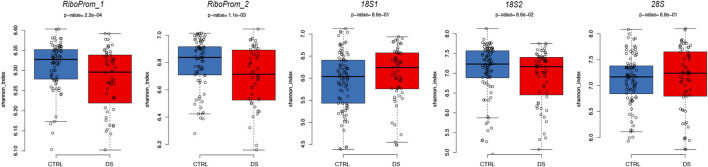
Epihaplotype diversity of the rDNA promoter in DS. For RiboProm_1, RiboProm_2, 18S1, 18S2, and 28S target regions, the boxplots report the Shannon diversity index in CTRL and DS. *P*-values were determined by the ANOVA test with correction for age, batch, and sex.

To further explore the DS-associated epigenetic alterations at the rDNA locus, we analyzed DNAm epihaplotypes. First, we calculated the Shannon diversity index, which is a measure of epihaplotype diversity within each sample. We then compared the Shannon diversity index between DS and control groups correcting for age, sex, and batch. We observed significantly lower epihaplotype diversity in persons with DS than that in controls (Figure 4) in *RiboProm_1* (*p*-value = 0.0002; q-value = 0.0011) and *RiboProm_2* (*p*-value = 0.0011; q-value = 0.0029). For *18S_1*, *18S_2*, and *28S*, no significant differences in epihaplotype diversity were found (Supplementary Table S3).

### Increased rDNA Methylation Variability in Persons With DS

**FIGURE 5:**
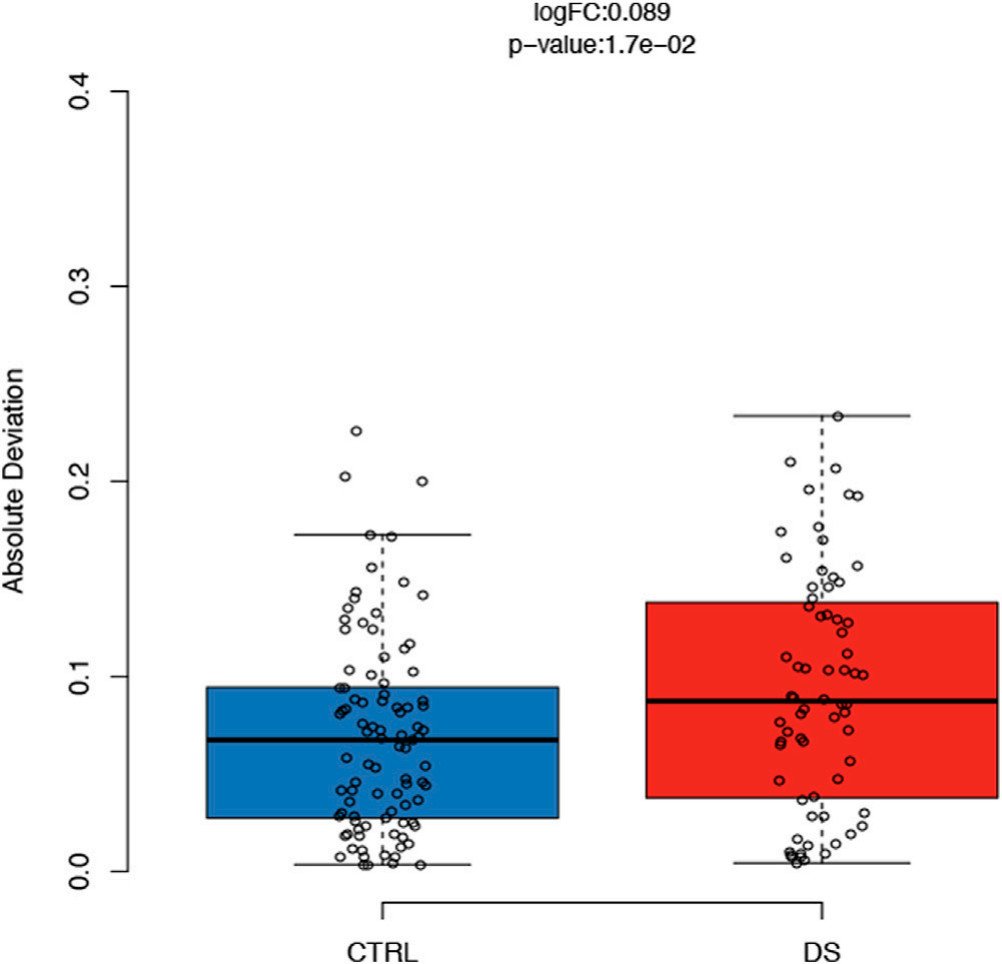
DNAm variability of the rDNA promoter in persons with DS. For CpG2 of *RiboProm_1*, the boxplots report absolute deviation values of DNAm in persons with DS and controls. LogFC and *p*-values were calculated using the R package *varFit.*

For each CpG site included in our assay, we compared the variability of DNAm values in DS and control groups after correction for sex, age, and batch. We found similar DNAm absolute deviation (AD) in the two groups for all the target regions except that for *RiboProm_1*. Indeed, most of the CpGs in *RiboProm_1* showed a significantly (*p*-value<0.05) higher DNAm AD in the DS group than that of controls (Supplementary Table S2), indicating that for this locus, persons with DS tend to be epigenetically more heterogenous than euploid subjects. Figure 5 reports DNAm variability for *RiboProm_1* CpG2 as a representative example.

### Reduction in rDNA Precursor Expression in Persons With DS

Finally, we evaluate whether the observed changes in rDNA methylation were correlated with the expression of the rRNA precursor RNA45S. At the time of recruitment, whole blood samples were not collected to preserve RNA integrity; we took advantage of PMBCs collected within the MARK-AGE project. Therefore, we analyzed RNA45S expression in PBMCs from 49 persons with DS and 33 euploid control subjects; for 42 and 29 of them, whole blood DNAm was measured (Table 1).

**FIGURE 6:**
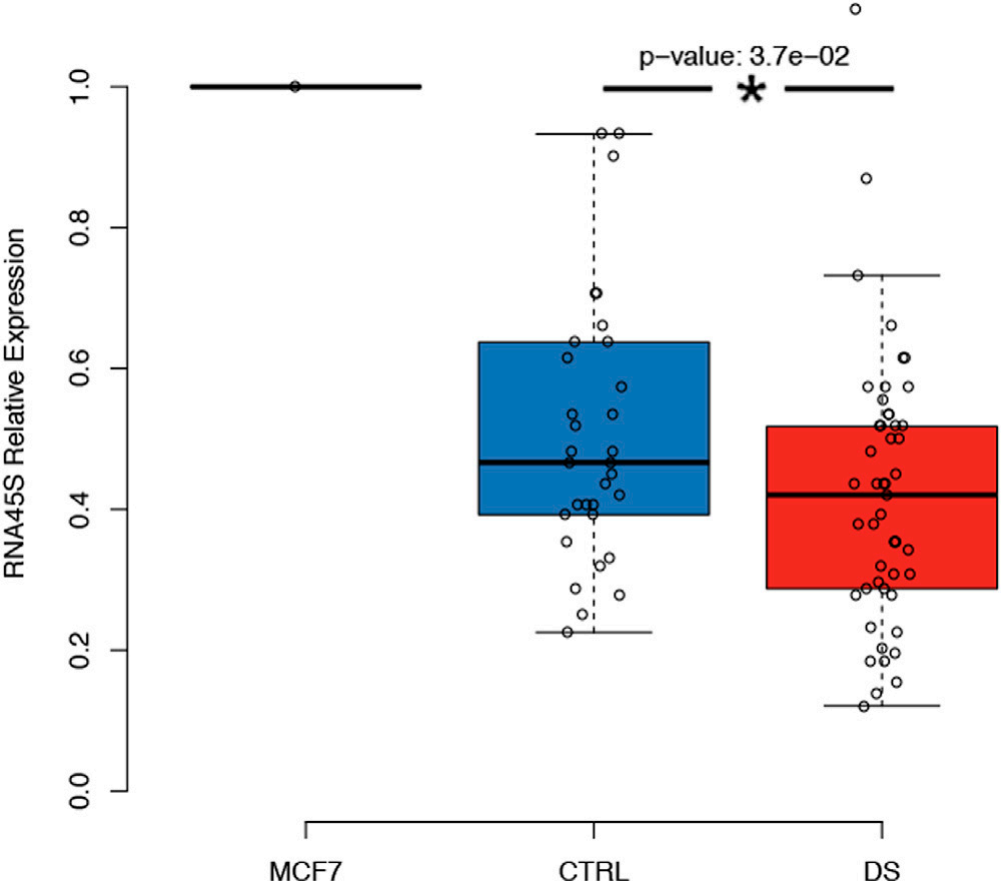
Expression of the RNA45S precursor in persons with DS. The boxplot reports the relative expression of RNA45S in CTRL and DS. RNA45S expression was measured using a standard **ΔΔCT** approach in which expression data were first normalized to an endogenous gene, the GUSB gene, and then to an internal calibrator consisting of an MCF7 cell line. *P*-value was determined by an ANOVA test with correction for age and sex.

We observed that RNA45S expression was significantly lower in persons with DS than in controls after correction for age, sex, and batch (*p*-value = 0.037) (Figure 6). The same result was obtained also when we excluded persons with DS younger than 35 years, as we did not have expression data for controls in this age range (Table 1; data not shown). We then analyzed the correlation between RNA45S expression and DNAm, considering DS and control groups separately (Supplementary Table S4; Supplementary Table S5, respectively). We did not observe any significant result except for CpG10 within *RiboProm_2*, for which RNA45S expression and DNAm were negatively correlated in controls. RNA45S expression did not show significant association with age neither in persons with DS (estimate = -0.0026, *p*-value = 0.248) nor in controls (estimate = 0.0058, *p*-value = 0.218).

## Discussion

In this study, we showed a trend toward hypermethylation of rDNA in whole blood cells from adults with DS compared to euploid controls. Hypermethylation of adjacent CpG sites tended to occur in all the regions evaluated within the rDNA unit (the promoter and the 5’ of 18S and 28S sequences), although it reached statistical significance mainly in the rDNA promoter. Albeit significant, the observed difference in DNAm tends to be small (around 3%), and its functional consequences, if any, should be further investigated. As methylation at the rDNA promoter is associated with the silencing of rRNA genes (Kresoja-Rakic and Santoro 2019), we can hypothesize that rDNA promoter hypermethylation in DS is the result of a compensatory epigenetic mechanism through which trisomic cells silence the extra rDNA copies in order to maintain the number of unmethylated rDNA units within physiological ranges (Lyapunova et al., 2017).

We also observed a reduction in the expression of the RNA45S precursor (a proxy of rDNA transcription) in PBMCs from persons with DS. It is possible that this phenomenon is not exclusively due to rDNA promoter hypermethylation as we did not observe a significant correlation between DNAm and expression in the group of persons with DS. It should be remembered that epigenetic mechanisms other than DNAm regulate rDNA expression and that inactive rDNA units are not transcribed despite not being methylated at the promoter (Kresoja-Rakic and Santoro 2019). Collectively, our results suggest that in adults with DS, there is a control over the transcription of rDNA and that DNAm can be one of the mechanisms involved in this process.

Our data are in partial, apparent contrast with previous reports that showed an increase in AgNOR staining in lymphocytes and buccal cells from infants and children with DS (0–12 years old) (Imamoglu et al., 2005, 2016; Imamoglu et al., 2006; Yilmaz and Demirtas 2008; Demirtas 2009) and suggested that an excess of active AgNOR is detrimental for *in utero* viability, accounting for about 10% of DS spontaneous miscarriages (Lyapunova et al., 2017; Porokhovnik and Lyapunova 2019). Demirtas et al. hypothesized that this excess in ribosome biogenesis occurring in the early phase of development causes energy waste and a general perturbation of cell metabolism, directly contributing to the DS phenotype (Demirtas 2009). Consistently with this view, increased ribosome biogenesis and enlarged nucleoli were described in fibroblasts derived from Hutchinson–Gilford progeria patients and old subjects (Buchwalter and Hetzer 2017; Phan et al., 2019) and are considered a hallmark of premature aging. This apparent discrepancy could be explained by the fact that we did not have infants with DS in our cohort. Indeed, a shift toward downregulation of ribosome biogenesis seems to occur in persons with DS after childhood (Borsatto and Smith 1996; Hamurcu et al., 2006). Lyapunova et al. (2017) evaluated the number of active ribosomal genes in lymphocytes from newborns with DS and older persons with DS (age range of 10–40 years) Although the mean number of active ribosomal genes was not different between the two groups, at older ages, there was an under-representation of trisomic subjects with an extreme (very high, but also very low) number of active NORs. The authors interpreted this result as the effect of selection against individuals with an abnormal number of active NORs that would have decreased viability and would, therefore, die in childhood. It is also possible that during the life of DS individuals, there is a progressive selection of cells in which abnormal ribosome biogenesis is controlled and reduced through different regulatory mechanisms that, as suggested by our results, can include DNAm.

DS is regarded as a segmental progeroid syndrome (Zigman 2013). Our data indicate that DS persons younger than 35 years tend to show DNAm levels of rDNA similar to that of older euploid subjects. However, caution should be taken in interpreting this observation as accelerated epigenetic aging of rDNA in DS, as the literature on age-associated DNAm changes of this locus is not consistent. An increase of DNAm of rDNA units, including the promoter, has been described in different tissues in mice, rats, and humans (Flunkert et al., 2018; Wang and Lemos 2019; Gensous et al., 2020b; Kerepesi et al., 2021; Shao et al., 2021). Conversely, D’Aquila et al. did not find age-associated changes in whole blood from individuals from 20 to 105 years. The discrepancy with our results can be explained by the different experimental approaches used as the group of CpG sites within RiboProm_2 that shows the most significant age-associated hypermethylation was not assessable by the EpiTYPER approach used by D’Aquila et al., (2017). However, our cohort has a smaller size and a narrower age range than the previously published one. It is worth to be noted that the hypermethylation of CpG_5 in the rDNA promoter was associated with lower cognitive performance and survival, and according to our assay, the same CpG was hypermethylated in DS. As a whole, our data support the idea that rDNA methylation in DS is set up at a higher level concerning euploid controls early during childhood and then remains stable with age. The analysis of epihaplotypes showed a significantly lower intra-individual diversity of *RiboProm_1* and *RiboProm_2* target regions in the DS group, meaning that persons with DS display a lower number of possible combinations of methylated and unmethylated CpGs along with the rDNA promoter. Consistently with what is described above, we can speculate that in cells from adults with DS, a tighter control over the epigenetic regulation of rDNA is in place in order to compensate for the extranumerary rDNA copies.

At the same time, we observed a larger interindividual variability within the DS group than that of the group of euploid subjects. This observation suggests that the outcome of the epigenetic regulation of rDNA is different among different persons with DS and fits with the large phenotypic heterogeneity observed among DS persons (Carfi et al., 2014). We attempted to investigate the possible biological meaning of this heterogeneity by correlating DNAm and expression values with neuropsychological data collected in the same cohort (Ghezzo et al., 2014), but we did not find any significant association (data not shown). Notwithstanding, it is interesting to note that increased heterogeneity is *per se* a characteristic of aging (BIOS consortium et al., 2016; Mahmoudi et al., 2019) and of pathological conditions (Zanin et al., 2018), and previous works showed higher variability of some molecular markers in DS (Conte et al., 2019). Intriguingly, the epigenetic status of rDNA repeats does not only regulate ribosome biogenesis but also affects the chromatin organization of the rest of the genome during development and cellular differentiation (Kresoja-Rakic and Santoro 2019). We can speculate that in DS, the presence of extranumerary copies of rDNA contributes to the global alteration of epigenetic and transcriptomic patterns that have already been described at early developmental stages (Vilardell et al., 2011). Further studies should verify this hypothesis and assess how the mechanisms regulating ribosome biogenesis (and possibly genome organization) are remodeled from development to adulthood in the presence of an extra copy of chromosome 21.

Our study has some limitations. First, changes in blood cell proportions between persons with DS and controls could affect DNAm measurements (Houseman et al., 2015), and we could not correct for this potential confounding effect as blood counts were not available for a large part of the control subjects.

Second, as rDNA repeats are genetically unstable and fragile in mammalian cells (Malinovskaya et al., 2018; Watada et al., 2020), we realize that rDNA copy number variation could also act as a potential confounding factor in our analysis. Furthermore, the assay that we used to quantify DNAm (based on the sequencing of short target regions within the rDNA unit) does not allow us to distinguish the multiple copies of rDNA units distributed on the short arms of the five acrocentric chromosomes nor to evaluate DNAm of the other CpG dinucleotides which locate within other regions of the rDNA unit. As a consequence, on one side, we were unable to determine whether rDNA hypermethylation interested all the rDNA repeats or only those located on chromosome 21; on the other hand, we could not exclude the involvement of other regions within the rDNA locus. Future studies using long-read sequencing technologies (Hori et al., 2021) could clarify both of these points.

The finding of a positive correlation between rDNA methylation and sequencing coverage is puzzling. Based on our experience and of a revision of the literature, an association between coverage and DNAm values has not been found or reported in other genomic regions (including highly repetitive regions such as Alu or LINE-1 sequences) (Flunkert et al., 2018). On the one side, it is possible that the observed association is the result of a PCR bias due to preferential amplification of target DNA depending on its original DNAm state. However, this explanation seems unlikely because five distinct target regions showed the same behavior and because the evaluation of DNAm standard curves did show a strikingly significant linear correlation between the observed and expected DNAm values. On the other side, it is possible that the observed effect is driven by biological differences between the samples, such as concomitant changes in rDNA copy number and methylation status. It should be noted that the PCR amplification of target regions is not quantitative but is likely to reach saturation, and therefore coverage cannot be regarded as a measure of rDNA copy number (and, indeed, coverage was not higher in DS than in CTR). However, it is worth to be noted that Roriguez-Algarra et al. recently showed a positive correlation between rDNA copy number and methylation of CpGs within the rDNA unit, assessed via whole-genome bisulfite sequencing (Rodriguez-Algarra et al., 2022), in agreement with our observation. Future studies should clarify the complex relationship between rDNA copy number and its methylation, taking into account the potential bias of the techniques used for their analysis.

Finally, the bisulfite treatment that we used in our experimental pipeline does not allow us to distinguish between DNA methylation and hydroxymethylation. Previous studies showed that DNA hydroxymethylation is altered in blood cells from persons with DS (Ciccarone et al., 2018), and the characterization of this epigenetic modification at rDNA units could provide additional information regarding the regulation of ribosome biogenesis in DS.

In conclusion, in this study, we reported that rDNA genes tend to be hypermethylated in the whole blood from adults with DS, suggesting that DNAm is one of the mechanisms involved in modulating ribosomal biogenesis in adults with DS. Further investigations are needed in order to shed light on the contribution of the epigenetic regulation of rDNA and, more in general, of ribosome biogenesis to DS pathogenesis.

## Data Availability

The datasets presented in this study can be found in online repositories. The names of the repository/repositories and accession number(s) can be found below: NCBI BioProject–PRJNA773620.
