# Supplementary figures and images for: DNA Methylation Analysis of Ribosomal DNA in Adults With Down Syndrome
Source: Front Genet. 2022 Apr 27;13:792165. doi: 10.3389/fgene.2022.792165 (PMC9094685; doi:10.3389/fgene.2022.792165)

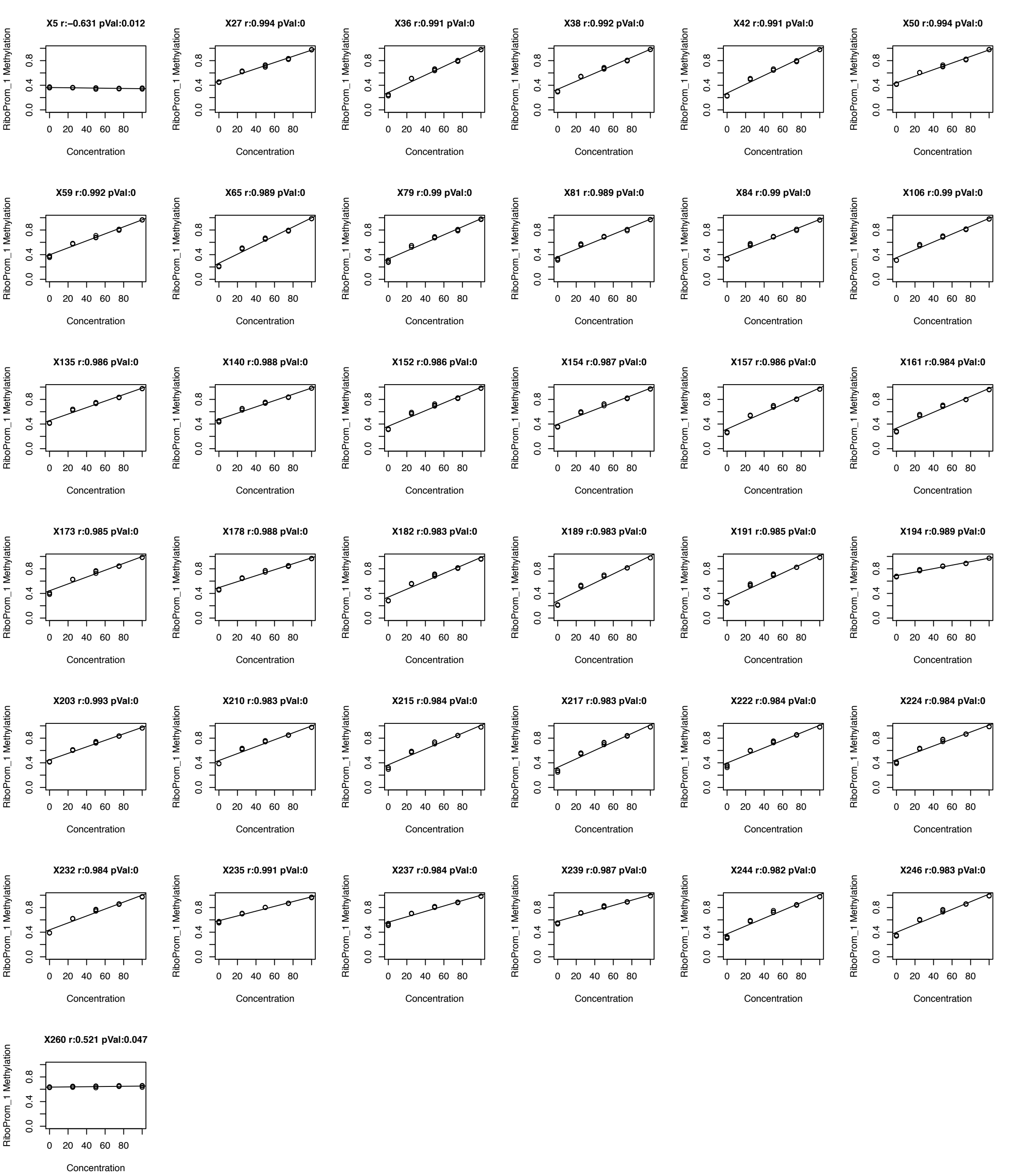

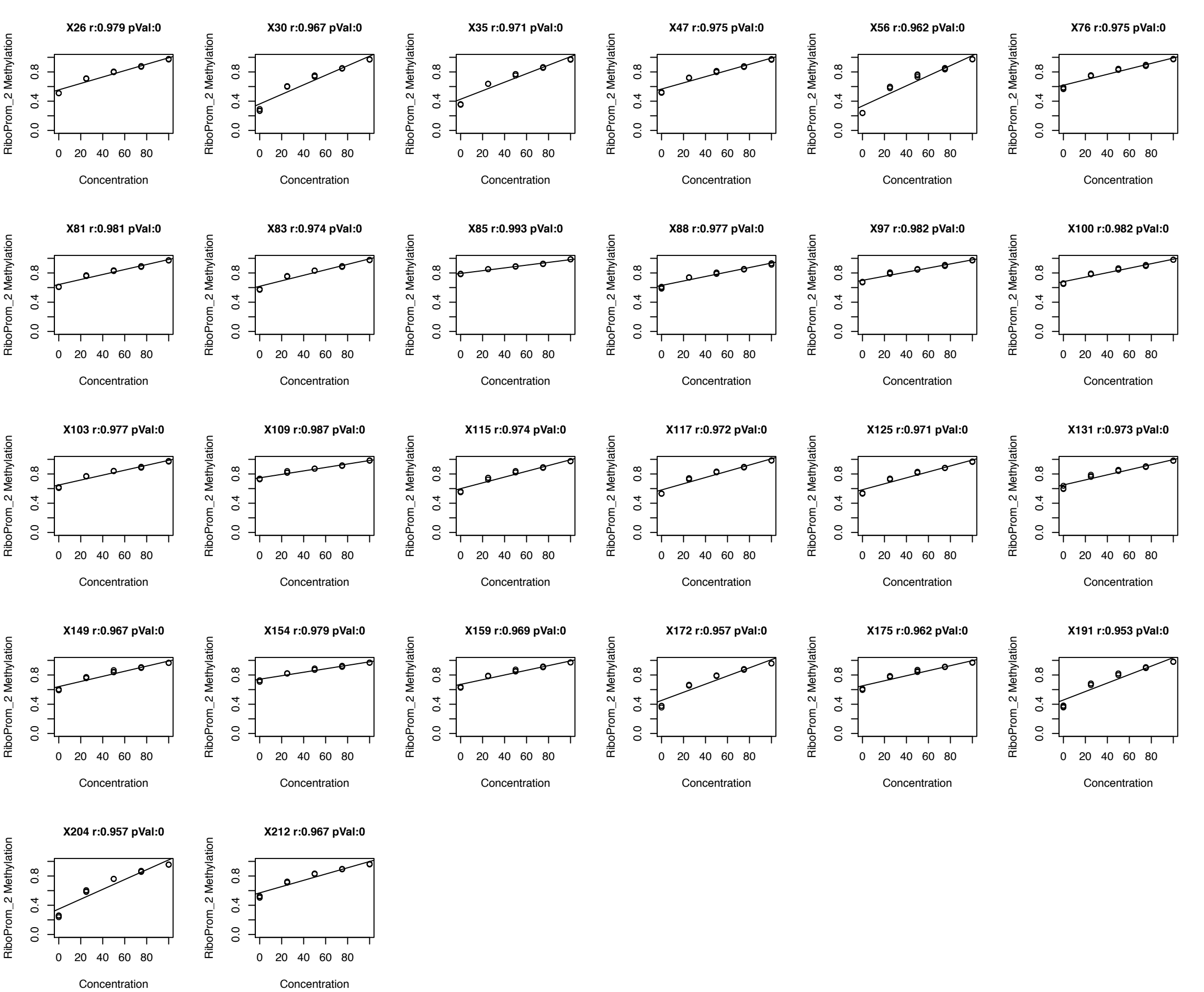

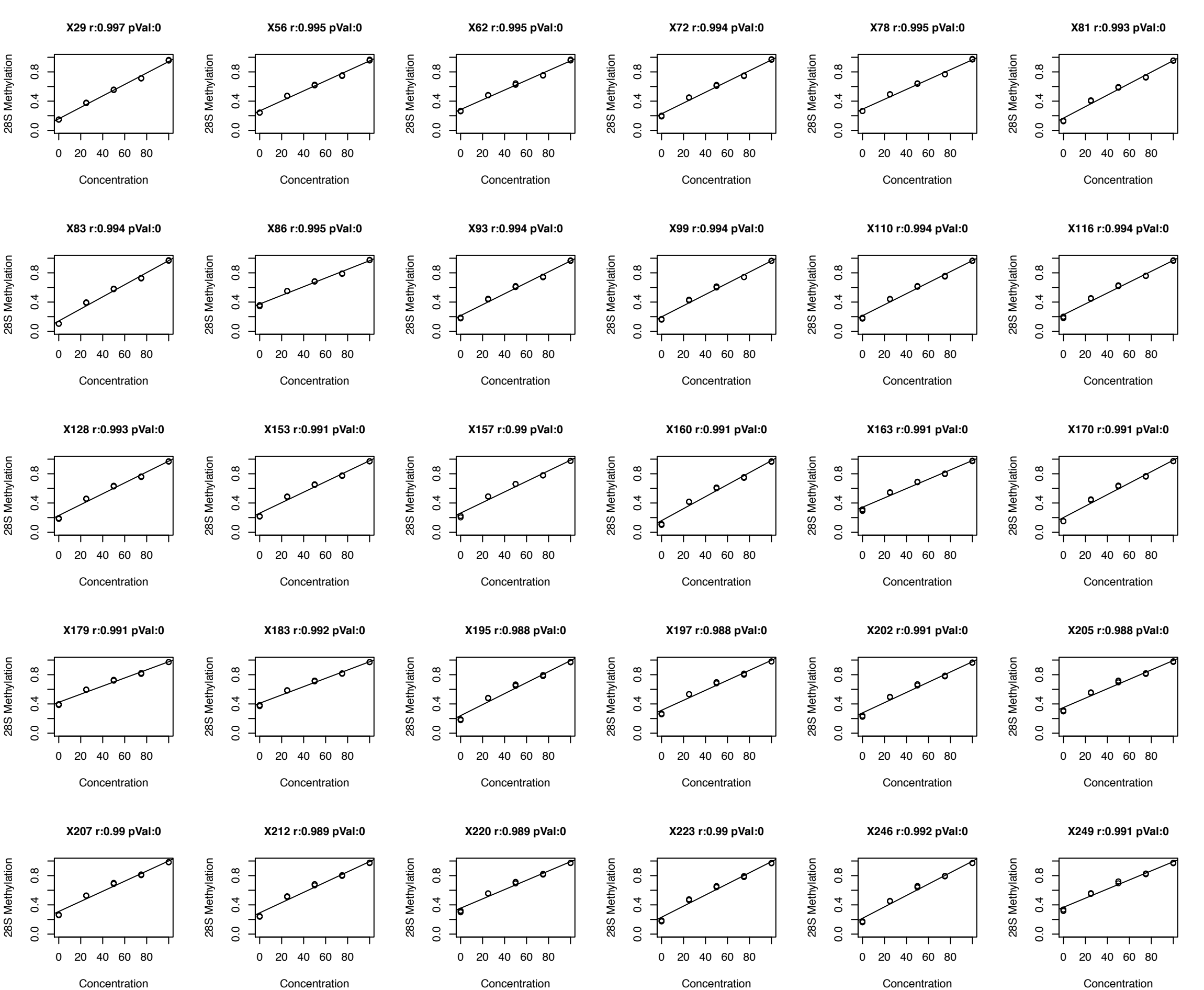

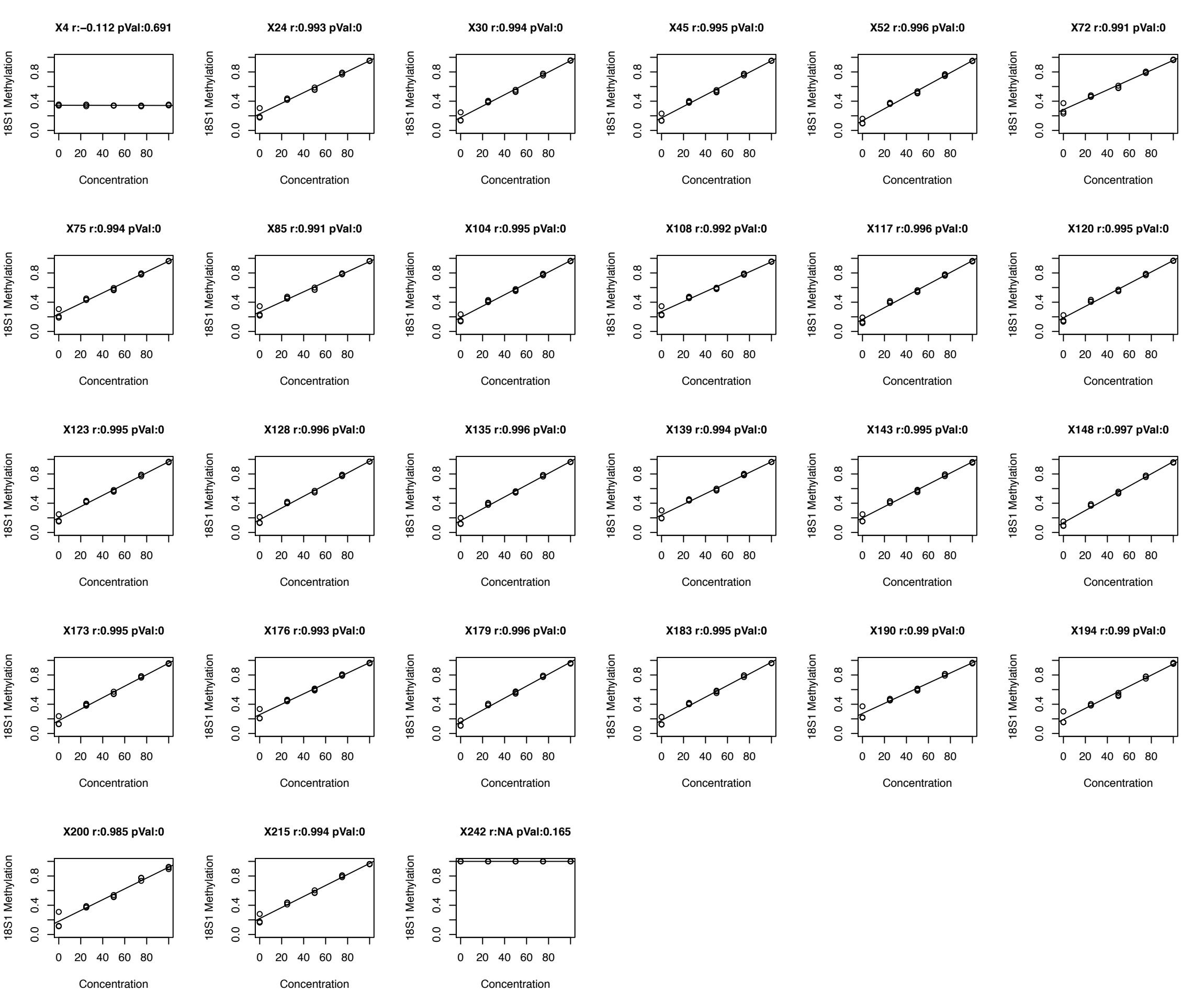

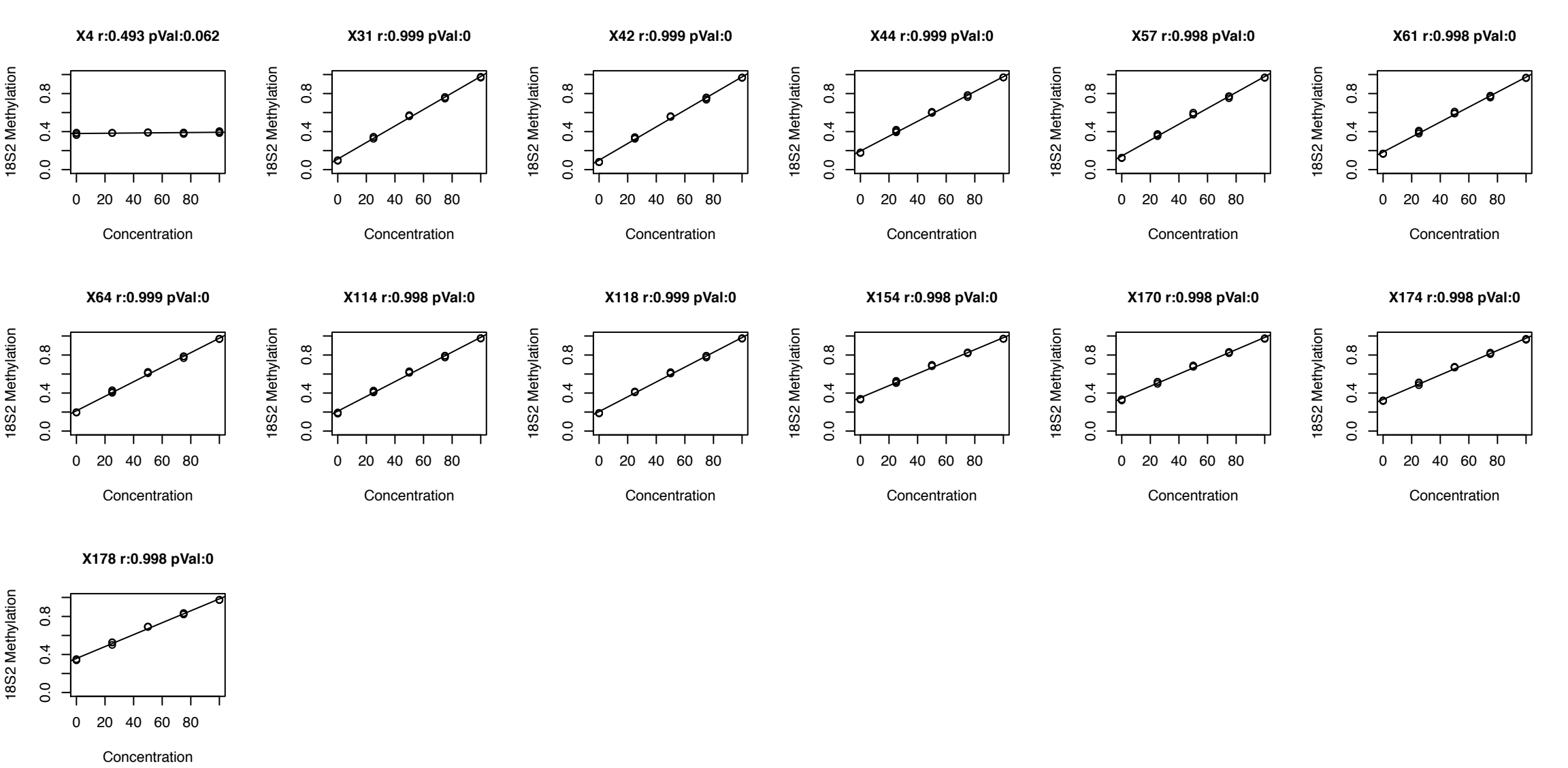

Supplement: Supplementary file 1 [file DataSheet1.zip › Supplementary Figure 1.PDF]

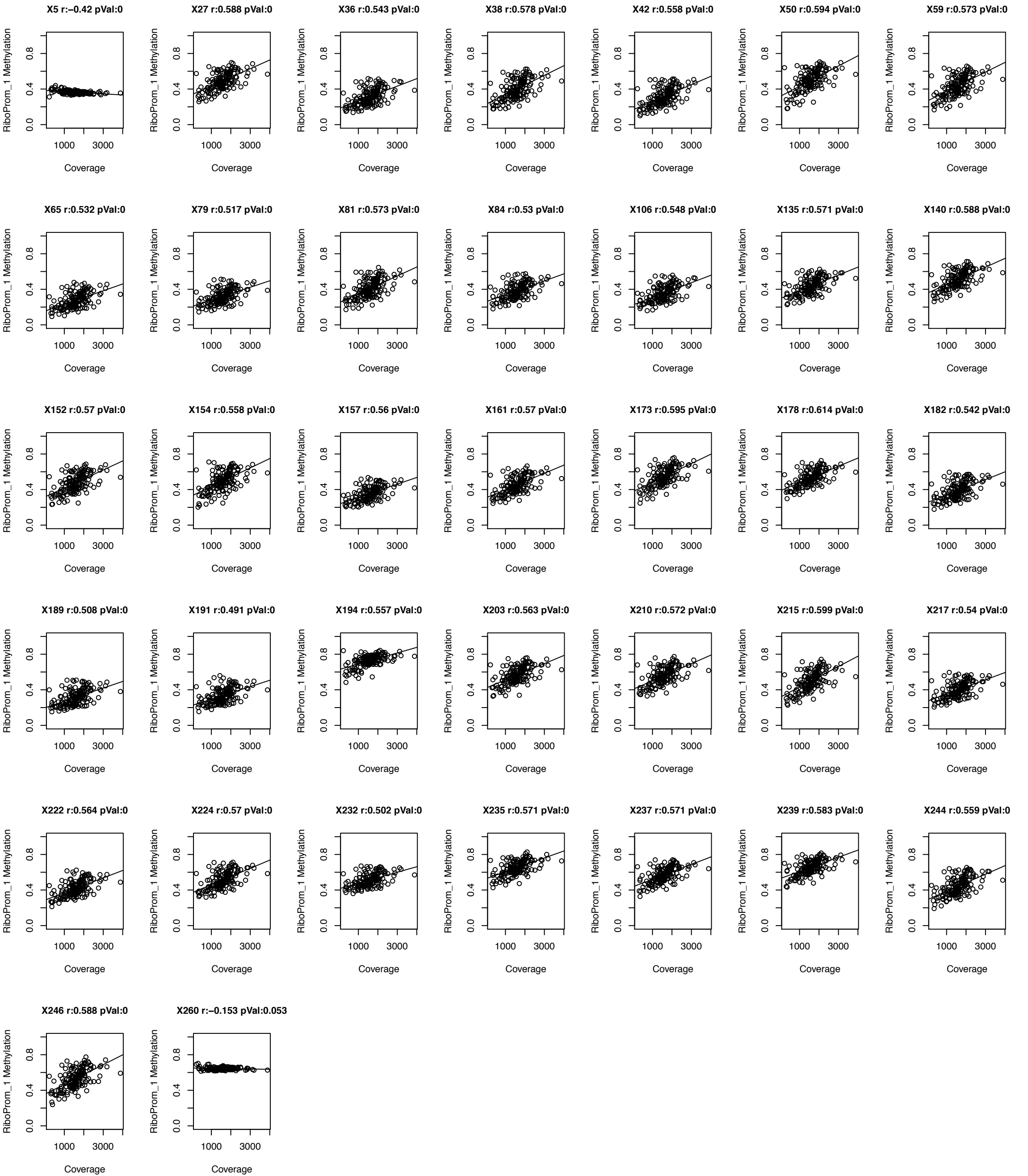

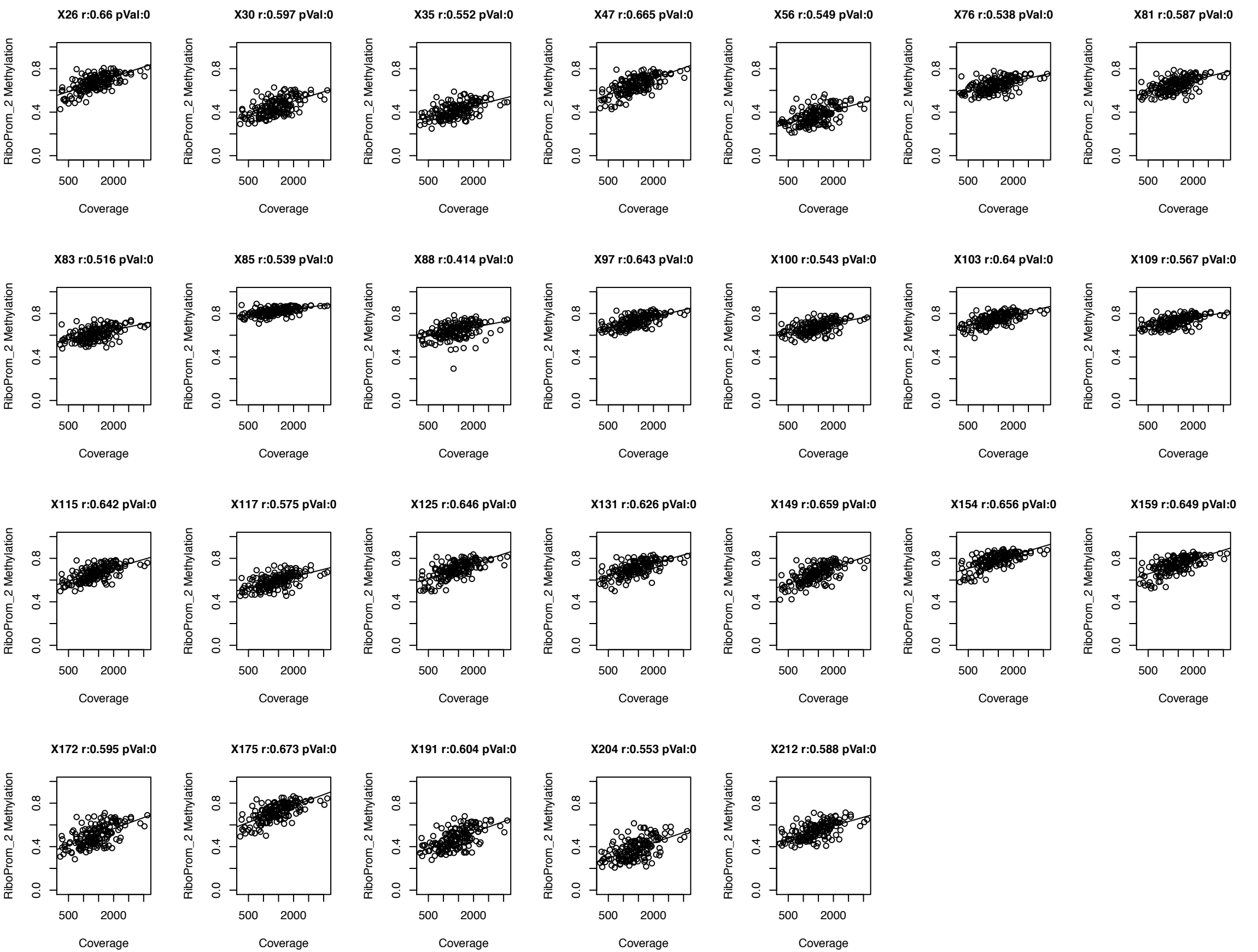

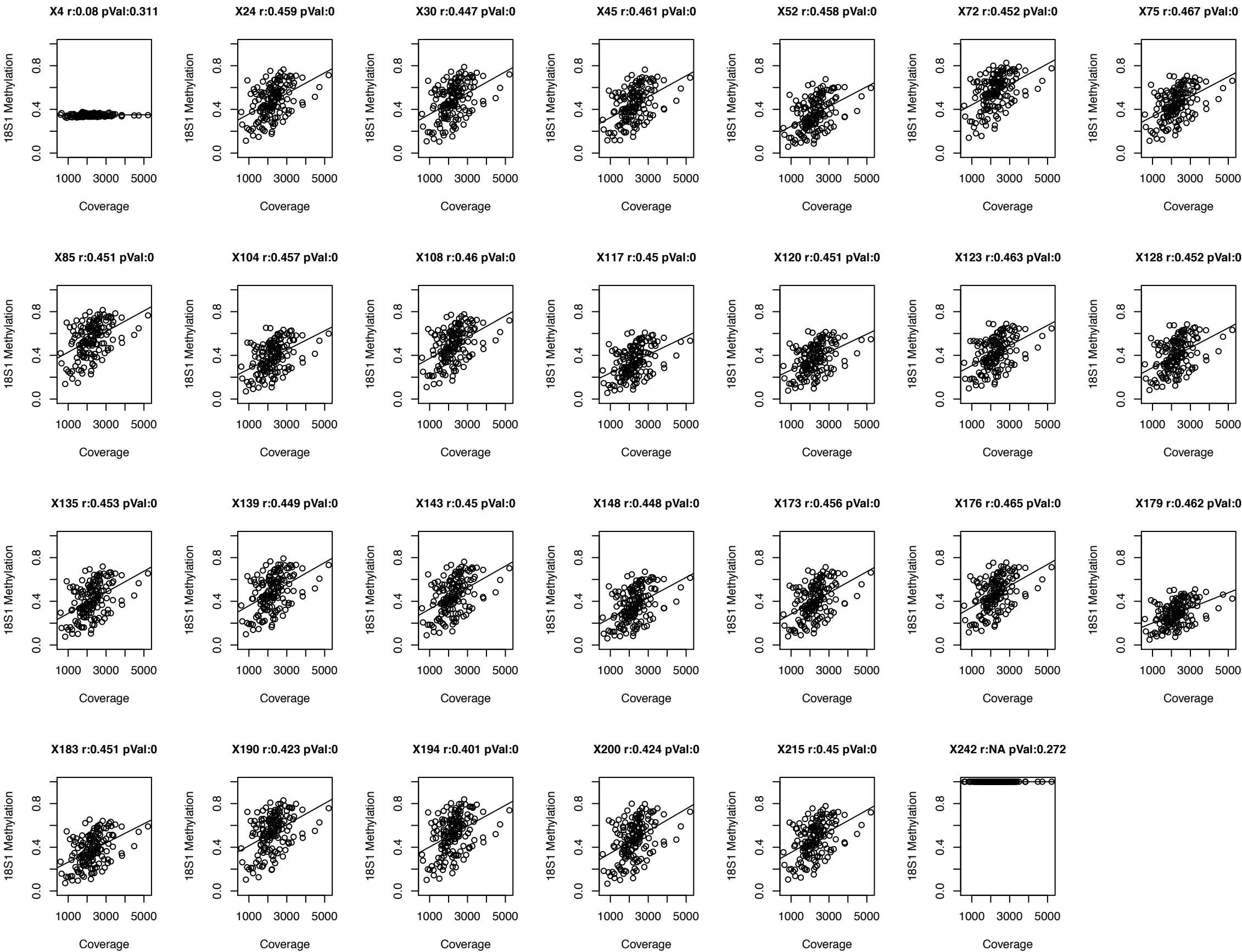

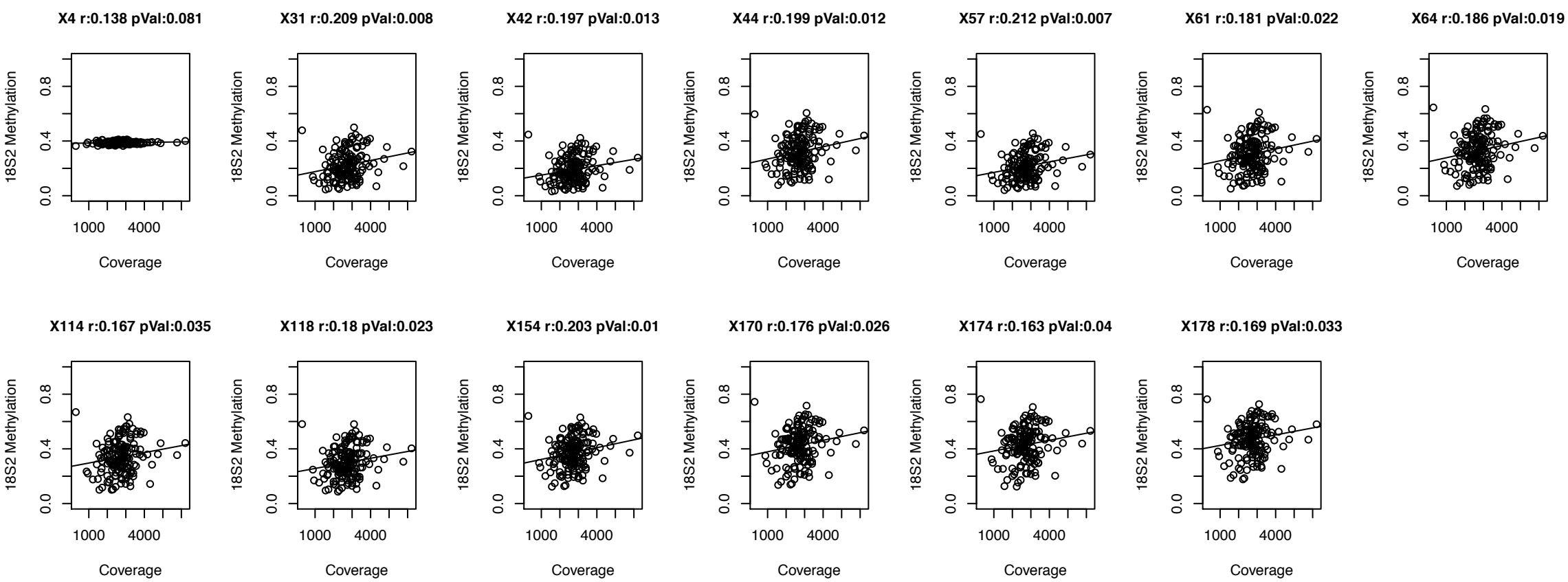

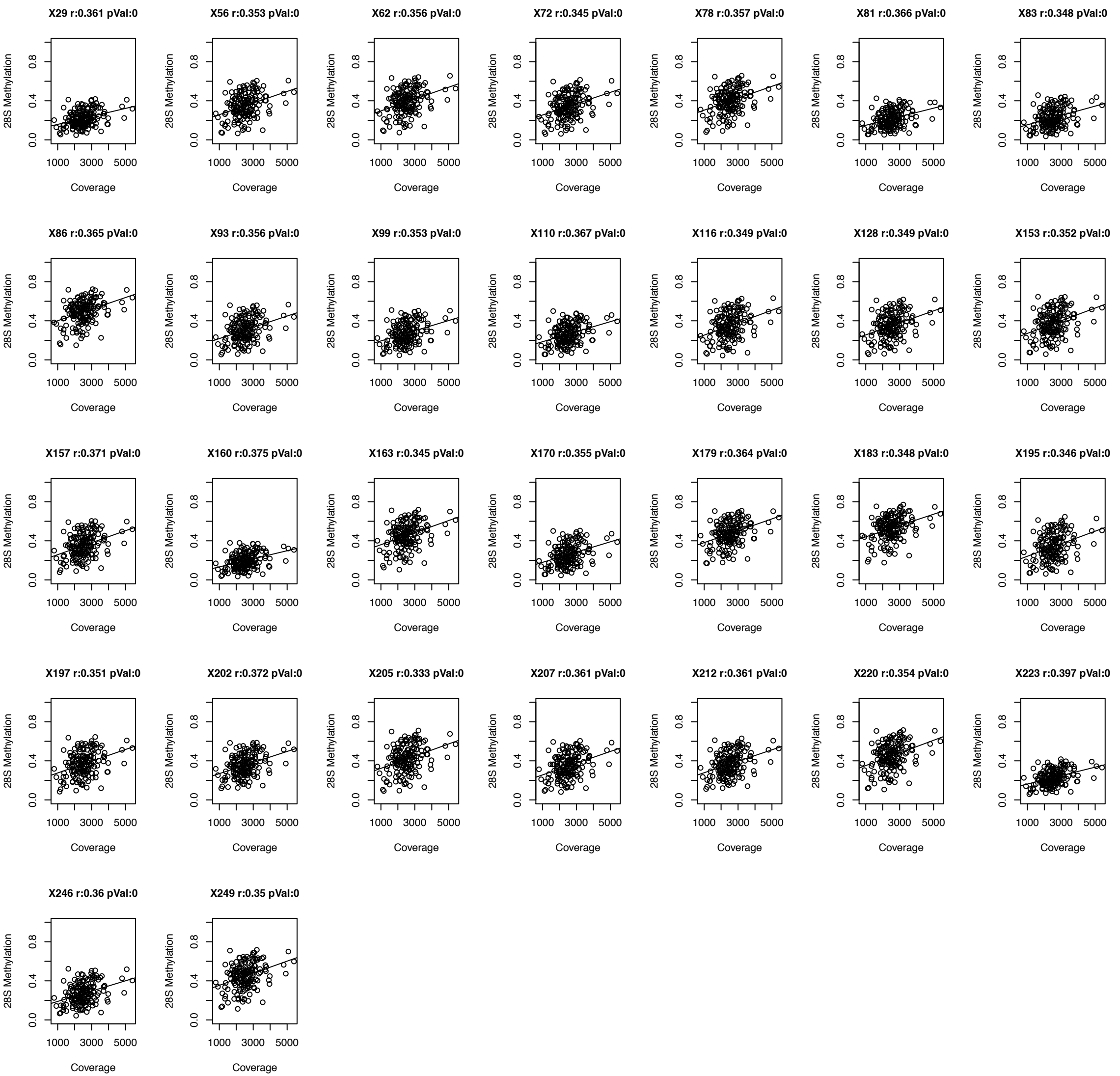

Supplement: Supplementary file 1 [file DataSheet1.zip › Supplementary Figure 2.PDF]

**RiboProm\_1**  
**p-value: 0.519**

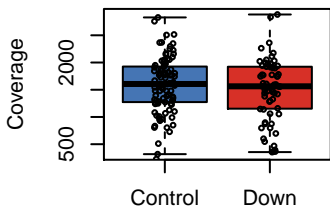

**RiboProm\_2**  
**p-value: 0.303**

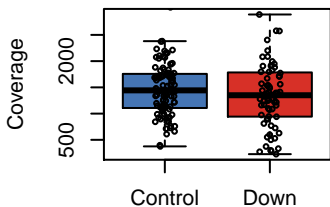

**18S\_1**  
**p-value: 0.108**

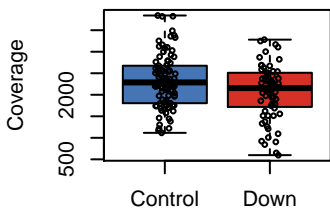

**18S\_2**  
**p-value: 0.003**

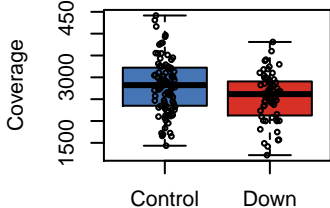

**28S**  
**p-value: 0.145**

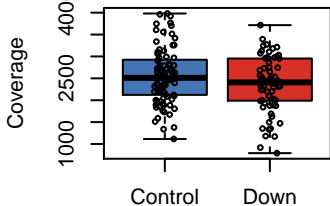

Supplement: Supplementary file 1 [file DataSheet1.zip › Supplementary Figure 3.PDF]

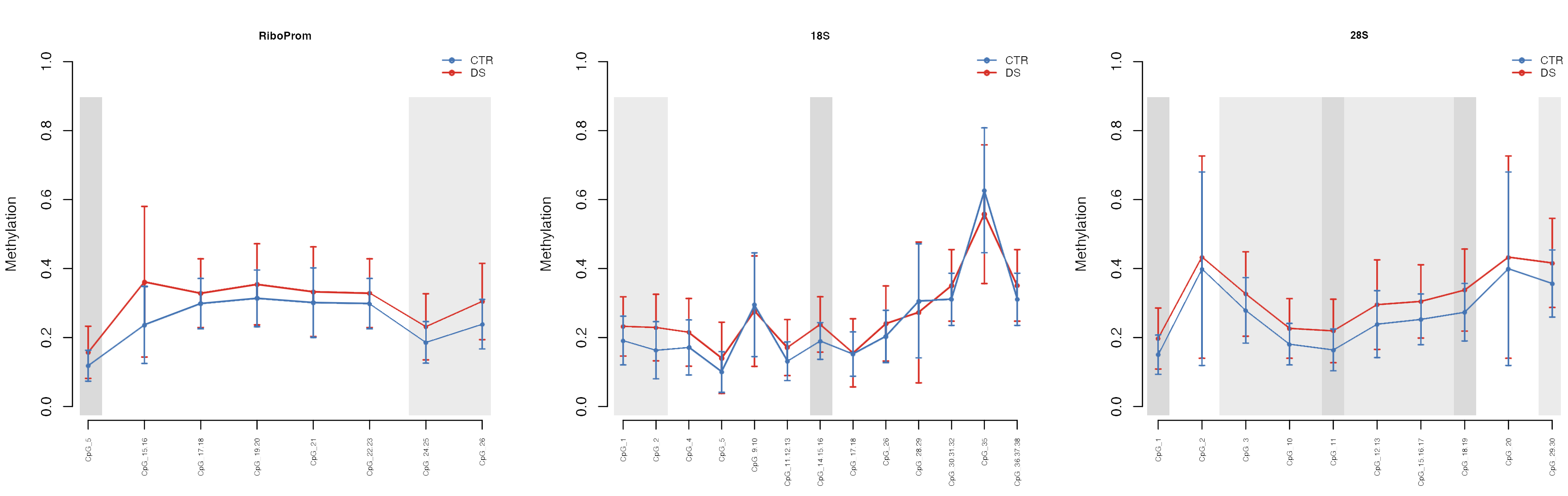

Supplement: Supplementary file 1 [file DataSheet1.zip › Supplementary Figure 4.TIF]
